# Clinical course of COVID-19 disease in immunosuppressed renal transplant patients

**DOI:** 10.3906/sag-2007-260

**Published:** 2021-04-30

**Authors:** Hamad DHEİR¹, Savaş SİPAHİ¹, Selçuk YAYLACI², Esma Seda ÇETİN², Ahmed Bilal GENÇ², Necattin FİRAT³, Mehmet KÖROĞLU4, Gürkan MURATDAĞI5, Yakup TOMAK6, Kezban ÖZMEN6, Ömer Faruk ATEŞ7, Oğuz KARABAY8

**Affiliations:** 1 ¹Division of Nephrology, Faculty of Medicine, Sakarya University, Sakarya Turkey; 2 Department of Internal Medicine, Faculty of Medicine, Sakarya University, Sakarya Turkey; 3 Department of General Surgery, Faculty of Medicine, Sakarya University, Sakarya Turkey; 4 Department of Microbiology, Faculty of Medicine, Sakarya University, Sakarya Turkey; 5 Department of Family Medicine, Faculty of Medicine, Sakarya University, Sakarya Turkey; 6 Department of Anesthesiology and Reanimation, Faculty of Medicine, Sakarya University, Sakarya Turkey; 7 Department of Radiology, Faculty of Medicine, Sakarya University, Sakarya Turkey; 8 Department of Infectious Diseases and Microbiology, Faculty of Medicine, Sakarya University, Sakarya Turkey

**Keywords:** COVID-19, SARS-CoV-2, kidney transplantation, immunosuppression, mortality, acute rejection

## Abstract

**Background/aim:**

We aimed to identify clinical settings of renal transplant patients with COVID-19.

**Materials and methods:**

In this retrospective study, we included kidney transplant inpatients with laboratory confirmed COVID-19 who had been discharged or had died by October 1st, 2020. Characteristics of the patients, including basal and last outpatient biochemical parameters were recorded. Discontinuation or dosage reduction of immunosuppressives and other treatment information was documented.

**Results:**

Twenty patients were included in this study, of whom 18 were discharged and 2 died in hospital. The mean duration of hospitalization and follow-up were 9.7 ± 6.4 days and 4.5 ± 2.0 months, respectively. Fourteen patients (70%) were male and mean age was 48.0 **±** 10.3 years. At admission, all had immunosuppression withdrawn and were started on methylprednisolone 16 mg/day (50%) or dexamethasone (50%). Tacrolimus/m-TOR inhibitors were reduced by 50% and all antimetabolites were discontinued. Hemodialysis was needed for 10% of patients. Acute kidney injury was detected in 25% of the patients. With respect to hospitalization time and complications, there was no significant difference between patients who used dexamethasone and those who did not (P > 0.05). The discontinued immunosuppressives were resumed within 2 to 4 weeks after discharge according to the severity of disease. No rehospitalization or acute rejection was detected during the follow-up of the patients.

**Conclusion:**

Renal transplant patients are considered a high risk group for COVID-19. It can be said that discontinuation or reducing dosages of immunosuppressives may be effective and safe in kidney transplant patients.

## 1. Introduction

The new coronavirus (COVID-19) outbreak is the worst disaster for humanity in the twenty-first century. The COVID-19 pandemic, caused by SARS-CoV-2 virus, has infected approximately forty two million people worldwide, and continues to pose a serious public health threat. COVID-19 disease may have a more fatal course when accompanied by certain comorbid conditions such as advanced age, diabetes mellitus, hypertension, heart disease, and solid organ transplantation [1]. Although renal transplant patients may have symptoms such as fever, cough, and shortness of breath, which are the most common symptoms observed during the course of the pandemic in the normal population, they may also have a broad range of atypical symptoms, such as fever and diarrhea without respiratory symptoms [2,3].

Short-term mortality rates due to SARS-CoV-2 pneumonia in patients with solid organ transplants are reported to be quite high compared to the normal population [4,5]. However, COVID-19 related mortality rates have been reported as 0%–41.3% and vary from country to country [6]. In addition, the populations of the reported studies in the literature were mostly cadaveric kidney recipients. Given their diminished T cell immunity, transplant recipients are expected to be at higher risk for severe bacterial and viral infections. Expected clinical course and mortality risk rates are higher in patients with renal transplants, due to administration of maintenance immunosuppressive agents and having comorbid conditions, compared to those of the normal population. So far, different approaches proposed for the renal transplant patients with COVID-19 include stopping antimetabolites and/or stopping or reducing the dose of calcineurin inhibitors (CNI) and increasing the dose of maintenance steroids [7,8]. However, although there is no clear evidence, some authors have suggested that CNIs may be used for therapeutic purposes for COVID-19 and there is no need for discontinuation in patients with renal transplants [9]. In addition to these treatment options, dexamethasone, hydroxychloroquine, remdesivir, favipiravir, lopinavir/ritonavir, tocilizumab, and convalescent plasma have also been reported as treatment options, although their exact effects still remain controversial [10–12]. However, while discontinuation of immunosuppressive therapy in these patients has an advantage in slowing the disease progression, there is a possibility that subclinical or clinical acute rejection attacks may occur by reactivating the immune system. In addition, there is no clear consensus yet regarding when discontinued or reduced immunosuppressive agents can be resumed.

The aim of this study is to describe the clinical features of kidney transplant patients with COVID-19 at the time of diagnosis and to investigate the patients and graft survival after treatment.

## 2. Methods

### 2.1. Study design and patients

This descriptive crosssectional study included 20 renal transplant patients treated for COVID-19 between March 20 and October 1, 2020. Demographic characteristics of the patients were recorded, including age, sex, body mass index, type and time of transplantation, immunosuppressive drugs used, presence of comorbid conditions, and the form of radiological involvement. The patient’s history of induction therapy, acute rejection therapy, and whether they had a viral infection were also documented. Symptoms, duration of complaint before admission, average hospitalization time, transition to the intensive care unit, or time of death were other parameters of interest. Baseline biochemical parameters and other items, including graft function tests, liver function tests, whole blood count, D-dimer, ferritin, lactate dehydrogenase (LDH), C-reactive protein (CRP), fibrinogen, prothrombin time, activated partial thromboplastin time, thoracic computed tomography (CT), and nasopharyngeal COVID-19 reverse-transcription polymerase chain reaction (RT-PCR) tests were used to determine the prognosis of the patients. RT-PCR tests were repeated within the first 24 h in patients with a negative first RT-PCR. 

### 2.2. Modification of immunosuppressive protocol

To control further progression of the underlying disease as well as for treatment purposes, antimetabolites were halted and CNI dose was reduced by 50% in patients with mild-to-moderate pneumonia (target CNI serum levels 4–6 µg/mL); receiving steroid/antimetabolite/CNI. If the patient was receiving steroid/CNI/mammalian or mechanistic target of rapamycin inhibitor (mTORi), it was planned to reduce the doses of both CNI and M-TORi by 50% (CNI level: 2–3, m-TORi level: 2–3 µg/mL). According to the severity of disease, it was planned to discontinue immunosuppressants, except for increasing methylprednisolone (16 mg/day) or switching to dexamethasone (6 mg/day) for 10 days. 

### 2.3. Statistical analysis

Data analysis was performed by using SPSS-22 for Windows (SPSS Inc. Chicago IL, USA®Z). The variables were investigated using visual (histograms, probability plot) and analytical methods (Shapiro–Wilk’s test) to determine whether or not they are normally distributed. We performed analyses to describe and summarize the distributions of variables. The continuous variables were expressed as mean and standard deviation or as median and interquartile range, depending on the normality of their distribution. The Mann–Whitney test was used to compare the variables that were not normally distributed. On the other hand, Student’s t-test was used to compare the variables with normal distribution. In two different periods of the disease, the Wilcoxon test was preferred to compare nonparametric variables, while paired Student’s t-test was used for variables with normal distribution. To compare the qualitative data, the chi-square test or Fisher’s exact test (when chi-square test assumptions do not hold due to low expected cell counts) was used. A P-value of < 0.05 was accepted as statistically significant.

## 3. Results

### 3.1. General characteristics

The study was carried out upon receiving the approval of the Ethics Committee of Sakarya University Faculty of Medicine (no.: 71522473/050.01.04/208 and dated 20.04.2020). Of the 380 renal transplant patients followed up at our center, 20 renal transplant patients were diagnosed with SARS-CoV-2. All the patients were hospitalized for treatment and follow-up. The most frequently detected blood group was blood group A with 45% amongst the patients. This was followed by blood group O with 30%, and blood group B with 25%. None of the patients had AB blood group type. Two patients (10%) died while the graft was functional because of acute respiratory distress syndrome due to severe COVID-19 pneumonia. After COVID-19 treatment, two patients had stage-5 chronic renal failure (CRF) and the patients were discharged to be treated with chronic hemodialysis (HD). The remaining 16 (80%) patients were discharged from the hospital after a full recovery.  

### 3.2. Clinical findings and hospitalization outcomes

The mean duration of hospitalization and follow-up were 9.7 ± 6.4 days and 4.5 ± 2.0 months, respectively. Of the patients, 14 (70%) were male and the mean age was 48.0 ± 10.3 (30–64) years. Median dialysis duration (IQR) before transplantation was 12.0 (3.75–45.75) months, and the mean time (± SD) from kidney transplantation to onset of COVID-19 infection was 71.9 ± 52.7 months. Prevalent comorbidities included hypertension (60%), chronic allograft dysfunction (15%), diabetes mellitus (25%), and chronic heart disease (15%). Their symptoms started at a median of 2 days (1–5 days) before admission to the hospital. The other demographic and biochemical parameters of the patients are summarized in Table 1. The most common symptoms were fever (75%), cough (75%), shortness of breath (50%), and myalgia (50%), whereas sore throat (15%) and diarrhea (5%) were less common. All patients were treated with anti-T-lymphocyte globulin (ATG) as induction therapy. Two patients were treated in the intensive care unit and the rest of the patients were treated in the ward. Of the patients, 85% had a history of living, and 15% deceased renal transplantation. In terms of complications developed during the treatment process, acute renal failure in 5 (25%) patients, sepsis in 2 (10%) patients, requirement for mechanical ventilation support in 2 (10%) patients, and a chronic hemodialysis program were required in 2 patients. Two patients who were included in the hemodialysis program were being followed up because they had stage 4 chronic kidney damage. Two (10%) patients died during the follow-up period (Figure).

**Table 1:** Demographic and baseline characteristics of kidney transplant recipients with a diagnosis of COVID-19.

Items	Results(n = 20)
Age (years), mean values ± SD (min–max.)	48.0 ± 10.3 (30–64)
Sex (F/M), n (%)	5/14 (30/70)
Body mass index (BMI, kg/m2)	25.5 ± 2.7
Blood groups, n (%) A B O AB	9 (45) 5 (25) 6 (30) -
Comorbid condition Hypertension Diabetes mellitus Chronic heart disease Chronic allograft dysfunction	12 (60) 5 (25) 3 (15) 3 (15)
Duration of dialysis prior transplantation (months), (IQR)	12.0 (3.75–45.75)
Transplant duration (months), mean values ± SD	71.9 ± 52.7 (7.2–157.8)
Mean time of hospitalization (days), mean ± SD	9.7 ± 6.4 (0–23)
Transplant type (%) Living (%) Deceased (%)	17 (85) 3 (15)
Bilateral/unilateral/normal chest CT findings (%)	15/3/2 (75/15 /10)
Change in immunosuppression, n (%) Discontinued antimetabolite only Discontinued antimetabolite and CNI/m-TORi Increased steroid dose	20 (100) 7 (35) 17 (85)
Supporting/antiviral treatment, n (%) Favipiravir Hydroxychloroquine Dexamethasone Oseltamivire Convalescent plasma Antibacterial antibiotics	18 (90) 10 (50) 10 (50) 9 (45) 6 (30) 13 (65)
Restarting time of immunosuppression, weeks	2.0 (2.0–3.0)
Duration of follow-up, months, mean values ± SD	4.5 ± 2.0 (0.7–6.5)

**Figure:**
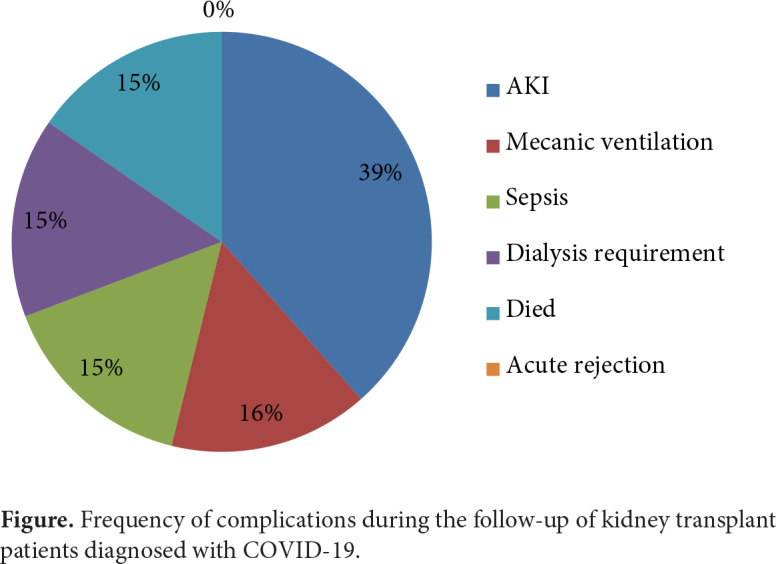
Frequency of complications during the follow-up of kidney transplant patients diagnosed with COVID-19.

The abnormal parameters detected at admission were significantly improved in the outpatient controls. During follow-up, no rehospitalization, acute graft dysfunction or acute rejection was detected in any of the patients. The mean resuming time of antimetabolites and CNIs was 2.0 (2.0–3.0) weeks. We resumed immunosuppressives after 2 weeks for patients with mild symptoms, and after 4 weeks for the patients with moderate or severe radiological involvement. 

### 3.3. Laboratory results

We identified RT-PCR positivity in all (100%) cases. Of these patients, 23% was confirmed to be positive with the second nasopharyngeal swab. Sixteen patients (90%) had bilateral typical COVID-19–associated radiological findings. The laboratory findings reported at the time of the diagnosis of COVID-19 infection were compared with those of the last outpatient control date. The median (IQR) values of basal serum creatinine was 1.15 mg/dL (0.93–1.83) and 1.13 (0.85–1.84) at the end of follow-up (P = 0.897). Similarly, a significant improvement was detected in white blood cell count (WBC) (P = 0.030), CRP (P = 0.001), alanine aminotransferase (P = 0.036), serum albumin (P = 0.018), and ferritin (P = 0.018) levels (Table 2). No treatment-related QT prolongation was detected in any of the patients. According to hospitalization time, complications like AKI, sepsis, needing mechanic ventilator, and mortality, there was no significant difference between the patients who used dexamethasone and those who did not (P > 0.05) (Table 3).

**Table 2:** Laboratory findings and comparison before and after COVID-19 infection treatment.

	Laboratory findings		
Variables	Outcomes at admission no = 16	Outcomes at outpatient no = 16	P-value
White blood cell count, 103/mm3	5.67 ± 2.77	7.88 ± 2.10	0.030
Absolute lymphocyte count, 103/mm3	1.22 ± 0.52	2.37 ± 1.37	0.319
Neutrophil to lymphocyte ratio	3.34 ± 1.96	2.75 ± 2.05	0.397
Platelet count, 103/mm3	211 ± 69	220 ± 87	0.006
Serum creatinine, mg/dL	1.15 (0.93–1.83)	1.13 (0.85–1.84)	0.897
C-reactive protein (CRP), mg/L	31 (20–58)	4.3 (3.0–18.2)	0.001
Procalcitonin, ng/mL	0.076 (0.031–0.48)	0.025 (0.1–0.03)	0.020
Alanine aminotransaminase, IU/L	25 (18–33)	18 (15–27)	0.036
Fibrinogen, mg/dL	449 ± 123	290 ± 77	0.570
D-dimer, ng/mL	325 (216–706)	2 32 (180–320)	0.173
Albumin, g/L	3.55 (3.3–4.17)	4.25 (3.6–4.6)	0.018
Ferritin, ng/mL	221 (102–795)	47 (20–136)	0.001

**Table 3:** Comparison of complications developed according to dexamethasone treatment.

		Using dexamethasone no = 10	Not using dexamethasoneno = 10	P-value
AKI	No	8 (80%)	7 (70%)	0.606
	Yes	2 (20%)	3 (30%)	
Sepsis	No	8 (100%)	8 (80%)	0.477
	Yes	0 (0%)	2 (20%)	
Need for hemodialysis	No	9 (90%)	9 (90%)	1
	Yes	1 (10%)	1 (10%)	
Mechanical ventilator requirement	No	10 (100%)	8 (80%)	0.474
	Yes	0 (0%)	2 (20%)	
Mortality	Deceased	1 (10%)	1 (10%)	0.702
	Alive	9 (90%)	9 (90%)	
Hospitalization (day)		5.5 (5–13)	9.5 (5–13)	0.673

### 3.4. Imaging results

According to thoracic CT findings, 15 patients (75%) had bilateral and 3 (15%) had unilateral lung involvement, whereas two patients (10%) with nasopharyngeal swab RT-PCR positivity had normal radiological findings. There were ground-glass opacities in 18 cases (90%), ground-glass combined with consolidation in 8 cases (40%), and bilateral pleural effusions combined with ground-glass image in 5 cases (25%).

### 3.5. Treatment protocols

Of the patients, 100% received 5 mg of maintenance prednisolone, 90% tacrolimus, 35% everolimus, and 85% was receiving mycophenolate mofetil (MMF). According to severity of the disease, prednisolone dosage was increased (16 mg/day) or started dexamethasone (6 mg/day) for 10 days, tacrolimus/mTORi dose was withdrawal or reduced by 50% and antimetabolites were discontinued in all patients. In addition, simultaneously favipiravir (90%), dexamethasone (50%), hydroxychloroquine (50%), oseltamivir (45%), and convalescent plasma (30%) were administered to the patients. Upon intense vomiting complaints in addition to fever in one patient, cranial MRI showed signs of temporal encephalitis. In this patient, everolimus treatment was discontinued, and prednisolone 20 mg/day and favipiravir treatment was started. Herpes meningitis was ruled out by taking a sample from cerebrospinal fluid. After a decrease in complaints and a full recovery, the patient was discharged.

Tacrolimus itself is rarely known to prolong QT [13]. No QT prolongation has developed in any of the patients using hydroxychloroquine and tacrolimus.

## 3. Discussion

In our study, we evaluated the clinical course, treatment approaches, and graft functions of kidney transplant patients with COVID-19 disease during the mid-term follow-up. During the COVID-19 pandemic, it is not easy to determine the appropriate treatment strategy in terms of patient and graft survival of transplant patients infected with SARS-CoV-2. Supportive care remains the mainstay of treatment for COVID-19, and there are currently no antiviral therapies with proven efficacy. Maintenance or increasing the dosage of steroid, withdrawal of antimetabolites, and reduction of the dosage of CNIs was considered in patients with COVID-19 [5,9–12]. In our study, we increased methylprednisolone or started dexamethasone therapy for all patients depending on the severity of the disease. Simultaneously, we also increased the dose of tacrolimus and resumed antimetabolites on the 4th week for the mild-severe cases and after 2 weeks in patients with mild-moderate findings. During the follow-up period we did not observe any acute rejection attacks after halting MMF and decreasing the dose of tacrolimus/m-TORi. However, discontinuation of immunosuppressive agents could hypothetically exacerbate inflammation in the absence of antiinflammatory agents. To our knowledge, acute rejection attacks were reported in patients diagnosed with COVID-19 in only one published case series [14]. 

Since hydroxychloroquine treatment was recommended at the beginning of the pandemic, half of our patients were treated with hydroxychloroquine. However, recently, it has been proven by observational study that hydroxychloroquine treatment is not effective for treatment or prophylaxis of COVID-19 disease [15]. One of the interesting antivirals suggested for COVID-19 treatment is favipiravir. It is recommended for patients with COVID-19, based on data suggesting efficacy against non-COVID-19 diseases [16]. Favipiravir has been shown to demonstrate a more efficient and rapid viral clearance in COVID-19 patients when compared to other antivirals [17]. However, there is no evidence-based randomized controlled study on the anti-COVID-19 efficacy of favipiravir. Another effective agent in the treatment of COVID-19 is dexamethasone, which has been proven to significantly reduce 28-day COVID-19-related mortality in the normal population [11]. In our study, we used dexamethasone treatment in transplant patients with moderate to severe disease severity. However, with respect to hospitalization time, complications and mortality, there was no significant difference between the patients who used dexamethasone and those who did not.

Of the patients, 90% were discharged from the hospital after a short period of hospitalization. One of two patients who died was a 64-year-old male patient who had a history of multiple comorbid conditions (diabetes, coronary artery disease, and peripheral artery disease, chronic graft dysfunction, and hypertension). He died from severe acute respiratory distress syndrome. The other patient who died had only hypertension comorbid status and died on the 16th day of admission. Thus, our mortality rate was as low as 10%. In a similar study of 10 cases (average age of 45 years), the mortality rate was reported as 10% [18]. In one study [5], however, intubation and mortality rates in transplant patients were as high as 39% and 28%, respectively, after 3 weeks of follow-up. The main reason for high mortality in this study may be the fact that the population was older (average age was 60 years), 75% had deceased transplant, and high ratio of comorbidities. In addition, two patients who were followed at home with mild symptoms died at home. Similarly, in a study of 20 renal transplant patients in Italy, COVID-19–associated 7-day mortality and intensive care unit hospitalization rates were found as 25% and 20%, respectively [19]. Publications of similar series are reviewed and summarized in Table 3. The mortality reported in the literature was higher than our findings [5,20,21]. There could be various explanations for that: most patients being transplanted from a cadaver, being older, having multiple comorbidities, or newly transplanted cases that eventually led to higher exposure to immunosuppression. However, our patients were transplanted from a living donor in 85% of the cases and the duration of developing COVID infection from the time of transplant was 71.9 ± 52.7 months. Therefore, we had lower comorbidity rates than other reported studies, where the baseline tacrolimus levels were below 6 ng/mL and the patients were younger. 

When we evaluated the common results of similar studies, factors such as advanced age, hypertension, and diabetes mellitus in transplant patients were found to be associated with high mortality rates. This suggests that stopping the antimetabolites and reducing the dose of CNI agents from the beginning may be useful in these patients. However, factors such as duration of transplantation, type of transplantation, acute rejection history, emergence of donor specific antibodies, advanced age, graft dysfunction, degree of radiological involvement of the lung, presence of sepsis, and comorbid conditions should be considered before deciding to discontinue or reduce immunosuppressive doses in these patients.

It has been shown that D-dimer level of >1 mg/mL elevation is a poor prognostic indicator of COVID-19 disease in the normal population [22]. However, in a multicenter study, no significant relationship was found between elevated D-dimer and mortality in kidney transplant COVID-19 patients [21]. We did not find a significant difference with respect to D-dimer levels between admission and outpatient in our patients. It may be due to the fact that our patient population has less comorbid conditions, received living kidney transplantation, and used steroid therapy.

Procalcitonin is a peptide released in the setting of systemic inflammation, in particular bacterial infections, and the magnitude of elevation has correlated with infection severity. Normally, procalcitonin is undetectable in the circulation. With the recognition that procalcitonin is a marker for severe infection, and has potential to differentiate bacterial infections from viral infections [23]. Especially in patients with severe COVID-19, a positive correlation was detected between elevated cytokines and procalcitonin levels [24]. In the follow-up of our patients, the progression of procalcitonin levels was detected in 65% of patients, and antibacterial treatment of the patients was initiated.

The present study has several limitations. The small sample size was the major limitation of our study. 

In conclusion, kidney transplant recipients may be at high risk of developing critical COVID-19 illness due to chronic immunosuppression and comorbidities. In our study, COVID-19–associated mortality rate was lower than those in other published studies. The main reason of this outcome may be the characteristics of our population which was different from these studies. We did not observe any acute rejection findings after halting MMF and decreasing the dosage of tacrolimus. For restarting immunosuppressives in these patients, resuming antimetabolites, and increasing the dose of CNIs on the 4th week for the mid-severe cases, and after 2 weeks in patients with mild-moderate findings may be a preferable treatment approach. 
